# Effect of screen time duration on psychosocial and behavioral aspects in adolescents (10-19 years): A cross-sectional study

**DOI:** 10.6026/973206300221051

**Published:** 2026-02-28

**Authors:** Naikey Minarey, Gaurav Kumar Prajapati, Tushar Talhan, Ankit Tiwari, Dileep Dandotiya

**Affiliations:** 1 Department of Pediatrics, Sunderlal Patwa Government Medical College, Mandsaur, Madhya Pradesh, India; 2 Department of Pediatrics, Government Medical College, Seoni, Madhya Pradesh, India; 3 Department of Psychiatry, Chhindwara Institute of Medical Sciences, Chhindwara, Madhya Pradesh, India; 4 Department of Pediatrics, Index Medical College and Hospital, Indore, Madhya Pradesh, India; 5 Department of Community Medicine, Chhindwara Institute of Medical Sciences, Chhindwara, Madhya Pradesh, India

**Keywords:** Screen time, mobile phones, psychological, behavioral

## Abstract

Adolescents have extensive use of screens and they have common complains related to mental health. Here a systematic review was done to understand the association between screen time and adolescent's mental health. Therefore, it is of interest to assess the effect of duration of screen time on psychosocial and behavioural aspects in adolescent children among 300 randomly selected children at the Department of Paediatrics, Index Medical College, Hospital and Research Centre, Indore. Data were collected from both inpatient and outpatient departments of the hospital using the strengths and difficulties questionnaire and their screen time. Most adolescents had personal gadgets (30.3%) or used those owned by close family members. A majority (78.3%) reported daily screen time between 1-5 hours, while 21.7% spent 6-10 hours daily on screen-based activities. Psychological outcomes highlighted alarming levels of distress, with 67.7% exhibiting depressive symptoms and 79.7% showing some form of anxiety. Behavioral issues such as irritability (34.3%), aggression (22.7%) and withdrawal (16.7%) were also prevalent. Thus, show that both the duration and content of screen use play a pivotal role in shaping mental health outcomes, making it imperative for caregivers, educators and policymakers to take a proactive role in moderating screen habits.

## Background:

Ranging from the age of 10 to 19, these shifts mark an inflection point for an individual; it is an emotional roller coaster drawing from psychological, mental and physical wheels of change [1]. There is a fluid development during this phase and from the age of transitioning from childhood to adulthood an individual's rational thinking, emotional control and social relations undergo through dramatic changes [2].This in fact informs the focus of concern for a nebulous period for an onset of serious health issues with the rise of anxiety disorder or depressive syndrome around this age [3]. Downright disturbing tendencies in the mental wellness of teenagers have been recorded by multiple works with the onset of chronic vulnerabilities like depression, anxiety or behavioral problems becoming more prevalent in recently observed patterns [4]. An alarming factor contributing towards this worrying trend is the excessive screen time especially of adolescent youth. Compared to other age groups, teenagers have taken to sedentary lifestyle with the introduction of smartphones, social networks and video gaming posing serious concerns for their psychosocial and behavioral health [5]. Increased screen time has a huge impact on screen time. It has been documented that excessive use of screens may lead to multiple physical, psychological and social challenges [6]. Physically, spending long periods of time on screens is connected to a number of obesogenic health issues, like poor sleep; sleep disorders, eye strain and headache disorders. Psychologically, increased screen time is strongly associated with greater anxiety, depression and other mental health problems [7]. The engaging character of digital content, especially social media can exacerbate negative body image, social isolation and feelings of inadequacy in young people. This is troubling, particularly because social relationships and interactions with peers are critical during this stage of development. For instance, online interaction could replace physical interaction, hindering the young person's ability to acquire appropriate and healthy instrumental and interpersonal social skills and meaningful relationships.

In addition, social media's exposure to idealized images can lead users to make an unsustainable norm-based comparison which, in turn, promotes anxiety; low self-worth and depression [8].With regard to screen time, its social media engagement impacts students to the extent of stunting their academic performance and disrupting critical elements of schooling. An increase in digital devices has been associated with a shortened attention span, concentration difficulties and a decline in academic performance [9]. Adolescents who spend more time on screens, especially during their leisure time, may find juggling school and homework more challenging. The use of screens before bedtime can also lead to sleep deprivation by negatively affecting sleep quality and duration. Inadequate rest can disrupt mood regulation, cognitive abilities and school performance. Less sleep increases the likelihood of developing anxiety and depression among adolescents [10]. All of the above, coupled with the influence of screen time, demonstrate how the changes induced by personal factors such as sleep, diet and academic performance interact alongside mental health and well-being [11]. Therefore, it is of interest to evaluating the effect of screen time on the personal as well as behavioral changes in adolescents between the ages of 10 and 19 years.

## Objectives:

[1] To assess personal changes (sleep patterns, sleep schedules, dental health, dietary habits, scholastic performance, obesity, addiction, headaches and refractive errors) and Social changes (social quotient).

[2] To evaluate behavioural changes (anxiety and depression) using psychiatric assessment scales.

## Materials and Methods:

This prospective cross-sectional study was conducted at the Department of Paediatrics, Index Medical College, Hospital and Research Centre, Indore from July 2023 to December 2024. Data were collected from both inpatient and outpatient departments of the hospital. Our study included adolescent children aged 10 to 19 years who attended the OPD/IPD services at Index Medical College. A total of 300 children were randomly selected for participation.

## Inclusion criteria:

[1] Children aged between 10 to 19 years.

[2] Children who had access to electronic gadgets (personal or parental).

[3] Children whose parents/legal guardians provided written informed consent.

[4] Children without any known congenital abnormalities or comorbidities such as Hypoxic-ischemic encephalopathy (HIE), cerebral palsy, or other congenital syndromes.

## Exclusion criteria:

[1] Children below 10 years or above 19 years of age.

[2] Children without parental consent.

[3] Children with congenital anomalies or significant comorbid conditions.

[4] Children without access to electronic gadgets.

## Methodology:

After obtaining informed consent, eligible participants were enrolled. Each Subject underwent:

[1] Detailed History and Questionnaire Assessment: A structured proforma was used to record demographic data, duration of screen time and relevant personal and social history, as provided by both parents and children.

[2] Personal and Social Changes Assessment: Psychosocial aspects were assessed using tools based on the Social Quotient (SQ) and Developmental Quotient (DQ).

[3] Behavioural Assessment: Behavioural changes were evaluated using standardized

## Psychiatric tools:

O Patient Health Questionnaire-9 (PHQ-9) to assess depression.

O Generalized Anxiety Disorder-7 (GAD-7) to assess anxiety.

These evaluations were conducted in the Psychiatry OPD by trained professionals.

## Nutritional and anthropometric assessment:

Anthropometric measurements, including height, weight, Body Mass Index (BMI) and body circumference, were recorded to assess growth and adiposity. Dietary history was taken to evaluate nutritional status.

## Investigation parameter

[1] Psychosocial and developmental assessment (SQ/DQ).

[2] Behavioural assessment using PHQ-9 and GAD-7 scales.

[3] Nutritional status via anthropometric measurements and dietary history.

## Statistical analysis:

The collected data were entered and analyzed using SPSS version 27.0, under the supervision of a statistician. Descriptive and inferential statistics were applied to explore associations between screen time and psychosocial/behavioural parameters. For mean comparison independent sample t-test was applied. A p-value of < 0.05 was considered statistically significant.

## Results:

Most adolescents (51.7%) were exposed to social media between the ages of 5-10 years, with 41.7% starting between 11-15 years. Early exposure to digital content may have long-term implications on psychosocial development. Above table showing that majority of participants were from 5-10 years (51.7%), followed by 11-15 years (41.7) (Table 1). Mobile phones were the most frequently used device (70%), followed by video games (49%), television (50.7%), tablets (45.7%) and laptops (35.7%). This demonstrated a trend toward personal, portable screen-based gadgets among adolescents (Table 2). Screen time was significantly higher in those with moderate (mean = 4.89 hrs) and severe depression (mean = 4.78 hrs) compared to those without symptoms (mean = 3.94 hrs), with p<0.001. This suggested a positive correlation between screen exposure and depression severity (Table 3). Mean screen time increased with anxiety severity-highest in severe cases (4.87 hrs) and lowest in mild (3.83 hrs), with p = 0.009. This showed that excessive screen time may exacerbate anxiety symptoms (Table 4). Withdrawal (4.80 hrs) and irritability (4.34 hrs) were associated with higher screen time than those with no behavioral issues (3.86 hrs) (Table 5). All four cases of refractive error occurred in the 6-10 hour screen time group, with none in the lower exposure group. The association was statistically significant (p <0.001), pointing to a risk of visual strain and refractive changes with prolonged screen use (Table 6). Mean screen time was significantly higher (4.84 hrs) in those using gadgets while eating versus those who did not (3.08 hrs), with p <0.001. This pattern suggests habitual screen exposure even during meals (Table 7). Screen time was highest among those exposed to action games (4.70 hrs) and lowest in action movies (3.95 hrs), with significant differences (p = 0.015). Exposure to aggressive content likely contributed to prolonged screen use (Table 8). Adolescents with higher screen time (6-10 hrs) spent significantly less time outdoors (mean = 1.83 hrs) compared to the 1-5 hrs group (mean = 2.24 hrs), with p = 0.006. This reflected a displacement of physical activity by digital screen engagement (Table 9). There was no significant difference in screen time between users and non-users of educational platforms (p = 0.190), indicating educational use did not independently influence total screen hours (Table 10).

## Discussion:

In our study, the analysis of age-wise distribution revealed that a significant proportion of participants exposed to prolonged screen time belonged to the 10-13-year age group, followed closely by those aged 7-9 years. This trend aligns with the findings of Twenge and Campbell [12], who reported a steeper rise in screen usage and its association with diminished psychological well-being in early adolescents compared to younger children. Their population-based study (n=40,337) highlighted a correlation between increased screen time and reduced curiosity, emotional stability and self-control among children aged 10-13, especially with more than one hour of daily exposure. Similarly, Santos *et al.* [8] (2023) emphasized that adolescents who predominantly use smartphones during weekdays show greater psychological vulnerability, particularly in mental well-being and social function. In our research, we found that depiction and duration of the participants' screens showed that children who had screen time greater than 2 hours a day were at higher risk of behavioral and psychological issues like inattention and social avoidance. These results align with Eirich *et al.* [13] (2022) that reviewed literature and reported greater screen time to have high significant association with externalizing and internalizing problems among children below twelve years. Our work also reported sharp increases in irritability and hyperactivity in children exposed to rapid-paced digital content or violence, supporting Lissak [14] (2018) who noted disruptive effects of violent video games and other stimuli on dopamine pathways that results in ADHD like symptoms, emotional volatility and sleep disorders in children. Another critical insight from our tables was the mediating role of sleep disturbances and reduced parent child interaction in children with higher screen exposure. Guerrero *et al.* [15] (2019) reported greater engagement in screen-based activities specifically, video games and R-rated media-and less sleep, along with an increase in aggressive and rule-breaking behavior, which was partially attributed to disrupted sleep cycles. Additionally, our results showed that not only the quantity but the type of screen content such as social media and action-based gaming was associated with lower psychological resilience and increased internalizing behaviors like sadness, withdrawal and anxiety. This supports findings from Santos *et al.* [8] (2023), who noted that social media use on weekdays, especially among adolescent girls, was strongly linked with lower mental well-being and higher risk of depressive symptoms. In our sample, children who lacked structured routines and parental oversight exhibited a higher screen dependency, further amplifying behavioral issues. These findings point toward a multi-factorial influence-where content, context and caregiver roles intertwine-to shape screen time outcomes in children. Our study observed that excessive screen exposure, especially before bedtime or involving violent content, was associated with difficulty in emotional bonding, decreased resilience and more frequent outbursts, reinforcing the conclusions of Mougharbel and Goldfield [16] (2020). They reported moderate-to-strong associations between screen time and poor psychological well-being, including depression, anxiety and body dissatisfaction, especially among adolescents and female participants. Our findings are also aligned with Zhao *et al.* [17] (2018), who revealed that psychosocial problems were significantly mediated by reduced parent-child interaction, which explained up to 58.6% of prosocial behavior deficits. In our sample, lack of interactive time with caregivers and increased unsupervised media consumption were particularly pronounced in children showing low empathy and cooperation in group settings. These results collectively emphasize that screen use especially passive or violent content may displace vital social interaction, diminish empathy and impair self-regulation, contributing to psychosocial deterioration in younger age groups. In the current segment of our study, the data pointed to a strong link between increased screen time and multiple psychosocial concerns such as poor sleep quality, emotional instability and reduced self-regulation in children and adolescents. Our observations resonate with the findings of Zhao *et al.* [17] (2018), who documented that over 78.6% of preschoolers exceeded the recommended 1 hour/day of screen time and each additional hour significantly raised the risk for poor psychosocial wellbeing primarily mediated by reduced parent-child interaction. Similarly, our study showed that children spending more time with screens had poorer social adaptability and emotional cooperation, a trend that closely mirrors the outcomes of Twenge and Campbell [12] (2018), who reported that children using screens for over 1 hour/day demonstrated lower curiosity, emotional stability and task completion ability, especially among older adolescents.

Our findings in the next segment indicate a consistent relationship between screen time and multiple behavioral difficulties such as aggression, peer conflict and inattentiveness among children and adolescents. This trend parallels the results of Eirich *et al.* [13] (2022), who conducted a large-scale meta-analysis involving over 159,000 children and found that increased screen time showed small but statistically significant correlations with both externalizing (*e.g.*, aggression, attention deficits) and internalizing (*e.g.*, anxiety, depression) problems. These behavioral manifestations, as captured in our tables, particularly in those with higher daily screen use, were evident in symptoms like irritability, difficulty concentrating and poor peer interaction. Our results further emphasize how screen time affects behavioral control and interpersonal relationships in children and adolescents. Specifically, issues such as aggression, defiance and rule-breaking behavior showed a clear rise with higher screen exposure, particularly with more than 2-3 hours daily usage. These findings align with Guerrero *et al.* [15] (2019), who reported that higher engagement with screen content, especially violent video games and mature-rated content, was significantly associated with elevated aggression, somatic complaints and reduced sleep, all of which directly correlated with behavioral dysregulation. The next segment of our findings demonstrates a continued association between screen time and reduced emotional stability, especially in the form of mood fluctuations, anxiety and attention deficits. Children with extended screen exposure, particularly over 3-4 hours per day, showed notable symptoms such as nervousness, social withdrawal and difficulty concentrating. These trends strongly correspond with the conclusions drawn by Twenge and Campbell [12] (2018), who reported that adolescents with higher daily screen exposure (7+ hours) were over twice as likely to exhibit clinical features of depression, anxiety and poor emotional regulation. Moreover, our study found a distinct trend of lowered prosocial behaviors and increased internalized emotional responses, such as guilt and fearfulness, in children with prolonged screen time. This aligns with findings from Zhao *et al.* [17] (2018), who highlighted that parent child interaction one of the primary buffers of emotional development is significantly disrupted by excessive screen exposure, thereby increasing internalized emotional difficulties in preschool children. Our study consistently demonstrated a strong inverse relationship between increasing screen time and various facets of child mental health, including attention span, behavioral regulation and social engagement. These trends are echoed in the findings of Eirich *et al.* [13] (2022), whose meta-analysis of 159,425 children concluded that screen time had small but statistically significant associations with both externalizing behaviors (*e.g.*, aggression, inattention) and internalizing behaviors (*e.g.*, anxiety, withdrawal). Our tables showing elevated irritability, lack of focus and lower cooperation with peers in high screen-time groups mirror these findings almost identically. Our analysis of problem behavior syndromes such as defiance, poor emotional regulation and impulsivity among high screen users is aligned with outcomes described by Mougharbel and Goldfield [16] (2020), who synthesized 60 studies and found moderate evidence linking high screen time with poor psychological well-being and body image dissatisfaction, particularly among female adolescents. Our findings resonate with their observation that gender-specific vulnerabilities, screen content type and passive screen use (*e.g.*, watching videos versus interactive learning) can moderate mental health outcomes.

## Conclusion:

Our study reinforces the growing body of evidence that prolonged screen time is significantly associated with a wide spectrum of negative psychological, emotional and behavioral outcomes among children and adolescents. The findings highlight clear patterns linking excessive screen exposure with reduced attention span, emotional instability, social withdrawal, poor sleep and heightened behavioral disturbances such as aggression and irritability. These adverse outcomes appear to be mediated through complex interactions involving sleep disruption, decreased physical activity and weakened parent-child interactions. Our results consistently show that both the duration and content of screen use play a pivotal role in shaping mental health outcomes, making it imperative for caregivers, educators and policymakers to take a proactive role in moderating screen habits.

## Funding:

Nil

## Figures and Tables

**Table 1:** Distribution as per age of exposure to social media

**Age of Exposure to Social Media**	**Frequency**	**Percentage**
11-15 years	125	41.7
5-10 years	155	51.7
>15 years	20	6.7
Total	300	100

**Table 2:** Type of gadgets used

**Type of Gadgets Used**	**Frequency**	**Percent**
Laptop	107	35.7
Mobile	210	70
Video Games	147	49
Tablet	137	45.7
Television	152	50.7

**Table 3:** Association between daily screen time and depression symptoms

**Depression Symptoms**	**Mean daily screen time**	**Std. Deviation**	**P value**
Mild	3.8	1.911	
Moderate	4.89	0.798	<0.001
None	3.94	2.155	
Severe	4.78	2.043	

**Table 4:** Association between daily screen time and Anxiety Symptoms

**Anxiety Symptoms**	**Mean daily screen time**	**Std. Deviation**	**P value**
Mild	3.83	1.603	
Moderate	4.03	1.675	
None	4.3	2.171	0.009
Severe	4.87	2.382	

**Table 5:** Association between daily screen time and behavioral symptoms

**Behavioral Symptoms**	**Mean daily screen time**	**Std. Deviation**	**P value**
Aggression	4.04	2.003	
Irritability	4.34	1.913	0.048
None	3.86	1.886	
Withdrawal	4.8	2.1	

**Table 6:** Association between daily screen time and Refractive Error

			**Refractive Error (Yes/No)**		**Total**	**P value**
			No	Yes		
	5-Jan	Count	118	117	235	
		%	79.70%	76.90%	78.30%	<0.001
Screen Time	10-Jun	Count	30	35	65	
		%	20.30%	23.10%	21.70%	
		Count	148	152	300	
Total		%	100.00%	100.00%	100.00%	

**Table 7:** Association between daily screen time and Usage While Eating

**Usage While Eating**	**Mean daily screen time**	**Std. Deviation**	**P-value**
**(Yes/No)**			
No	3.08	1.459	<0.001
Yes	4.84	1.943	

**Table 8:** Association between daily screen time and Type of Aggressive Content

**Type of Aggressive Content**	**Mean Daily Screen Time (hrs)**	**Std. Deviation**	**P-value**
Action Games	4.7	2.998	
Action Games, Action Movies	4.43	1.732	
Action Movies	3.95	1.622	0.015
None	4.65	1.631	

**Table 9:** Association between daily screen time and mean Outdoor Activity Time(hrs)

**Screen time (in hrs)**	**Mean**	**Std. Deviation**	**P value**
5-Jan	2.24	1.065	0.006
10-Jun	1.83	1.039	

**Table 10:** Association between daily screen time and online education platform usage

**Online Education Platform Usage**	**Mean Daily Screen Time (hrs)**	**Std.Deviation**	**P value**
No	4	2.255	0.19
Yes	4.32	1.832	
